# Time Capsule Medicine: A Mixed-Methods Pilot Study on Immersive Simulation for Chronic Disease Education in Medical Students

**DOI:** 10.3390/clinpract15040078

**Published:** 2025-04-09

**Authors:** Andreas Conte, Waseem Jerjes

**Affiliations:** 1 Faculty of Life Sciences & Medicine, King’s College London, London SE1 1UL, UK; andreas.conte@kcl.ac.uk; 2 Faculty of Medicine, Imperial College London, London SW7 2AZ, UK; 3 North End Medical Centre, Hammersmith and Fulham PCN, London W14 9PR, UK

**Keywords:** chronic disease, medical education, simulation training, continuity of patient care, empathy, educational measurement

## Abstract

**Background**: Chronic diseases require long-term and multidimensional care, but traditional medical education has emphasised acute care and episodic interventions. This gap limits the understanding of future clinicians about the complexity of managing chronic conditions over decades. This mix-methods quantitative–qualitative pilot study describes “Time Capsule Medicine”, an innovative educational framework in which medical students acted out the progressive challenges that patients with chronic illnesses might face over a continuous period of 20 years. This paper aims to report the outcomes of this innovative educational technique. **Methods**: Thirty final-year medical students were engaged in the three-phase programme which included preparation, immersive simulation, and reflection and evaluation. The preparation consisted of online workshops in chronic disease progression, age-related changes, and continuity of care, while the immersive simulation featured appropriate role-play exercises in small groups that simulated the chronic disease process across four five-year increments. The reflection and evaluation consisted of debriefing sessions and reflective journals, while pre- and post-simulation questionnaires tested learning outcomes. The physical constraints included weighted garments with visual impairments simulating the age-related limitations. **Results**: A gender- and ethnically diverse cohort of thirty final-year medical students from three medical schools in North London participated in the programme. The simulation significantly enhanced students’ confidence in managing long-term disease trajectories (pre-simulation score: 2.8 ± 0.9; post-simulation score: 4.3 ± 0.6; *p* < 0.01) and understanding of age-related challenges (from 3.1 ± 1.0 to 4.5 ± 0.5; *p* < 0.01). Empathy scores increased from 3.0 ± 0.9 to 4.7 ± 0.5 *p* < 0.01. The qualitative analysis showed an increased appreciation of the continuity of care, recognition of systemic barriers, and insights into the emotional burdens of chronic conditions. For many students, the simulation was transformative, changing the way they approached holistic, patient-centred care. **Conclusions**: This experiential learning approach has succeeded in effectively addressing one of the most important gaps known in traditional medical education in developing empathy, understanding, and confidence in the long-term management of chronic diseases. The integration of similar simulations into medical curricula may adequately arm future clinicians with the complexities of continuity of care and patient management. Further studies need to be performed exploring scalability and its impact on long-term clinical practice.

## 1. Introduction

Chronic diseases are the leading burden of illness globally [1], and their management requires long-term, multidimensional care beyond isolated clinical encounters [2]. Traditional medical education often emphasises acute care and episodic interventions [3]; thus, future clinicians usually are underprepared for the management of the complexities of chronic conditions over decades [4]. These conditions too often involve progressive challenges in forms such as the loss of vision, restrictions in mobility, cognitive decline, and emotional burden [5], deeply shaping factors in the healthcare journeys of these patients.

The possibility of understanding the cumulative effect of these challenges is deeply rooted in the continuity of care, empathy, and a patient-centred approach [6]. Nevertheless, opportunities for medical students to gain first-hand insight into the long-term trajectories of chronic diseases remain limited [7]. Although traditional curricula may establish foundational knowledge [3], seldom do they consider the evolving interplay of physical, emotional, and social factors over the lifetime of a patient [8].

Role-play has been shown to be an effective teaching methodology for medical students and trainee doctors [9,10,11]. However, there has been little focus on facilitating medical student simulations of the lived patient experience. In rare cases, simulations have focused on the experience of ageing, with some evidence of improvements in medical student knowledge, attitudes, and empathy towards older people [12]. Crucially, to the best of the authors’ knowledge, there are no studies that have facilitated medical students’ simulation of chronic diseases, such as diabetes, osteoarthritis, and dementia [13].

In this context, “Time Capsule Medicine” is an innovative teaching methodology in which students simulate the natural 20-year development of a chronic disease. Students in medicine engage in elaborate role-playing to experience the incremental decline in conditions like diabetes, osteoarthritis, and dementia. This can create an opportunity for gaining great insight into the management of a disease over a long period of time, assist in the strategies adopted for holistic care, and help them build competency regarding the adaptation of clinical strategies for the patient’s changing needs.

This study had two primary objectives:

(1) To quantitatively evaluate the impact of an immersive, time-lapse simulation on medical students’ confidence, empathy, and understanding of chronic disease management, measured through pre- and post-simulation surveys.
(2) To qualitatively explore students’ lived experiences of managing chronic illness over time, including their reflections on the continuity of care, emotional and functional challenges, and systemic barriers to effective patient-centred care.

## 2. Materials and Methods

### 2.1. Study Design

This study employed a mixed-methods design that integrated quantitative and qualitative data to assess the impact of the Time Capsule Medicine simulation on knowledge of chronic disease management in medical students. The quantitative component followed a single-group, pre–post, and quasi-experimental design without a control group, using surveys administered before and after the intervention. The qualitative component used a phenomenological approach to explore students’ lived experiences during the simulation, based on reflective journals and debrief discussions.

The intervention was separated into three structured phases: (1) preparation, during which time students learned about chronic disease progression through workshops and baseline measurements; (2) immersive simulation, during which time students participated in a role-playing exercise of a 20-year chronic disease course; and (3) reflection and evaluation, during which time students completed debriefings, post-simulation questionnaires, and qualitative reflection. The structured nature of this design allowed for a thorough analysis of the effects of experiential learning on beliefs of chronic disease management in students (Table 1).

**Figure 1:**
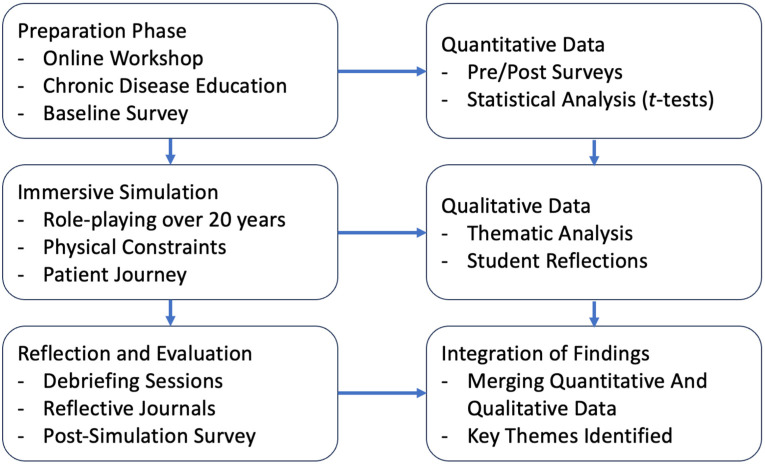
This figure outlines the structured phases of the study, demonstrating the integration of quantitative and qualitative methodologies.

To ensure a comprehensive evaluation, both quantitative and qualitative data were collected, analysed, and integrated across study phases to assess changes in confidence, empathy, and understanding of chronic disease management (Figure 1). The preparation phase involved online workshops on chronic disease education, followed by immersive simulation, where students engaged in role-playing over a simulated 20-year disease progression. The reflection and evaluation phase included debriefing sessions, reflective journals, and post-simulation surveys. Quantitative data were collected through pre- and post-simulation surveys and analysed using statistical methods, while qualitative data were derived from thematic analysis of student reflections. Findings from both data types were integrated to assess the educational impact of the intervention, providing insights into improvements in confidence, empathy, and understanding of chronic disease management.

### 2.2. Setting and Participants

The programme took place within structured educational settings, including dedicated simulation suites, and online platforms for preparatory workshops. The immersive role-playing exercises were facilitated in small-group settings, allowing students to engage in realistic patient scenarios over a simulated 20-year disease trajectory. Post-simulation debriefing and reflective activities were conducted in faculty-led discussion sessions, ensuring a structured review of learning outcomes. The study environment was designed to foster experiential learning without reliance on advanced technological simulations, emphasising the cognitive, emotional, and practical challenges of chronic disease management. For the qualitative component, a convenience sampling strategy was used, drawing data from all participating students who completed the reflective journals and post-simulation debrief discussions.

Thirty final-year medical students in three North London medical schools were enrolled in this study. The selection was achieved through an advert sent via email. Written consent with all the necessary information pertaining to the study was provided well in advance to all participants. These students were diverse in their clinical interests and experiences, with prior rotations in primary care, medicine, and surgery. Inclusion criteria required students to have completed at least one primary care or internal medicine rotation, while exclusion criteria included prior participation in similar immersive simulations or lack of consent.

A sample size of 30 students was determined based on pre-study statistical estimations, using an expected absolute risk reduction (ARR) of 40–60% and a Number Needed to Treat (NNT) range of 1.5–3.0 to detect meaningful improvements. Power calculations indicated that this cohort size would achieve statistical significance at *p* < 0.01, while ensuring feasibility within the study design. This approach aligns with established methodologies for evaluating educational interventions, allowing for a robust assessment of the simulation’s impact.

### 2.3. Variables and Data Sources/Measurement

The primary outcome variables measured in this study were student confidence, empathy, and knowledge related to chronic disease management. These were assessed using an ad hoc pre- and post-simulation questionnaire developed specifically for the simulation. The questionnaire included five-point Likert-scale items (1 = Strongly Disagree; 5 = Strongly Agree) to quantify levels of confidence, empathy, understanding of continuity of care, and recognition of non-clinical barriers. Scores were analysed as continuous variables, and changes were reported as mean score improvements. The questionnaire also included open-ended questions to capture narrative responses about expectations and perceived learning.

Since no existing validated instrument addressed the unique experiential aspects of this immersive simulation, the tool was reviewed by two medical education experts for face validity. Internal reliability was not formally assessed due to the pilot nature of the study but will be evaluated in future larger-scale studies.

Quantitative data were also collected using pre- and post-simulation surveys to assess student confidence, empathy, and knowledge of chronic disease management. Student confidence was assessed using a 5-point Likert scale, and empathy was assessed using guided reflection questions. The variables were compared using percentage improvement, mean scores, and standard deviations to ascertain the effectiveness of the intervention. Qualitative data were also collected using written reflection and debriefing discussions, which were thematically analysed to identify key learning points. The work did not involve using patient records or clinical data, as all measurement was through student-reported outcomes and simulated scenarios.

### 2.4. Intervention Structure

#### 2.4.1. Phase 1: Preparation

Participants attended a workshop that introduced the concept of “Time Capsule Medicine”. The overview given in this online workshop included the following:

- An in-depth overview on chronic diseases, such as diabetes, osteoarthritis, and macular degeneration, and how these conditions evolve over decades;
- Discussion of age-related changes: physical frailty, impairment of vision, and decline of cognition;
- There are ethical considerations in the understanding and empathising of the journeys of the patients by insisting on continuity of care.

Pre-simulation questionnaires were given to determine the baseline knowledge and attitudes of the students regarding the management of chronic diseases (Appendix A). These questionnaires used Likert-scale questions that measured the confidence in managing long-term conditions and open questions regarding the expectations of the exercise.

#### 2.4.2. Phase 2: Immersive Simulation

The simulation aimed to replicate the progressive challenges faced by patients with chronic illnesses over a 20-year period, broken into four five-year phases, each representing new stages of disease progression and their effects on daily life and healthcare interactions (Table 2):

- Year 0–5: Early-stage disease with minimal functional impact. Students simulated managing a new diagnosis and initiating medication.
- Year 6–10: moderate disease progression, introducing physical limitations, such as mobility challenges (e.g., using weighted clothing), and addressing intermittent symptoms and assistive device use.
- Year 11–15: Advanced stages with significant physical and emotional strain. This phase included simulations of vision impairments (e.g., blurred vision goggles) and the complexities of managing multiple medications.
- Year 16–20: Severe disease progression characterised by high dependency, cognitive impairments (e.g., memory challenges), and reliance on caregivers or structured medical routines.

During the simulation, students participated in role-playing exercises to emulate tasks that patients face, such as the following:

- Managing complex medication schedules with visual or cognitive limitations;
- Navigating to medical appointments despite physical constraints;
- Balancing daily activities, like cooking or personal hygiene, alongside health-related challenges.

Students documented their experiences at each stage, focusing on the physical, emotional, and practical difficulties encountered. The simulation emphasised reflecting on how these challenges could shape a patient’s healthcare journey over time.

#### 2.4.3. Phase 3: Review and Reflection

After the simulation, students participated in a post-simulation debriefing session facilitated by a clinical educator (Author: WJ). This activity allowed the discussion of observations and valuable insight gleaned from the simulation. The students reflected on how the complications unfolded and proffered strategies on how continuity and quality of care could be improved for patients with chronic illnesses.

Surveys were also distributed after the simulation to evaluate changes in knowledge, confidence, and attitudes. These questionnaires contained Likert-scale questions concerning students’ learning about long-term illness trajectories and open-ended questions to elicit qualitative responses.

Reflective journals were also gathered in which the students answered the following questions:

- “Describe the most significant challenge you faced during the simulation and, importantly, how it enhanced your insight into the management of long-term patient care.”
- “How did the simulated disease progression influence your concept of continuity of care?”
- “What changes would you recommend in the healthcare system to better support patients with chronic conditions over decades?”

### 2.5. Bias

Several measures were taken to restrict potential sources of bias in this work. Self-selection by those most interested in chronic disease management was restricted by inviting all eligible final-year medical students to participate. Response bias was prevented by making questionnaires administered pre- and post-simulation anonymous, to enable truthful self-reporting. Observer bias was prevented by using standardised questionnaires and guided reflection questions to enable consistency in collection of data. However, in that this work was self-reported in nature, there is a risk of social desirability bias, in that participants would have replied in a socially acceptable manner that they perceived, rather than one that was representative of their actual experience.

### 2.6. Analysis Process

Quantitative data from pre-and post-simulation questionnaires were analysed to determine a change in confidence and understanding related to the management of chronic disease. Means and standard deviations were calculated, whilst the percentage of students improving significantly was estimated using absolute risk reduction (ARR) and Number Needed to Treat (NNT). Statistical significance was established using paired t-tests, a significance of *p* < 0.01 was employed.

Qualitative data from reflective journals and group discussions following debriefing were thematically analysed following a phenomenological approach, which focused on identifying core themes related to students’ lived experiences of long-term chronic disease management. This was achieved through recording and transcribing participants speeches, independent coding by the two authors to establish categories of analysis, and a consensus analysis between authors to draw out key themes. Such a non-technological yet structured methodology provided rich insight into the long-term problems facing patients and the role of continuity in providing patient-centred care. The methodology assured a focus on experiential learning without dependence on advanced tools, with wide generalisability of findings to medical education.

### 2.7. Ethical Considerations

This study was conducted as a quality improvement project and classified as a service evaluation, aiming to enhance medical education on chronic disease management. As such, it did not require formal ethical approval under institutional guidelines. This study did not involve direct patient participation; instead, all activities were conducted in a controlled educational setting using structured role-playing exercises.

Participation in the study was voluntary, and all students provided informed consent before engaging in the simulation and subsequent data collection. Confidentiality was maintained by anonymizing all data, with participants assigned unique study identifiers to ensure privacy. No personally identifiable information was recorded, and survey responses were stored securely in compliance with data protection regulations.

Future iterations of the simulation could integrate patient narratives, testimonials, or recorded interviews to enhance realism and provide first-hand insights into the lived experiences of managing chronic conditions. Such additions would complement the simulation by deepening students’ understanding of the emotional, social, and systemic challenges faced by patients, further reinforcing the importance of continuity of care and patient-centred management.

## 3. Results

### 3.1. Participant Demographics

A total of 30 final-year medical students from three North London medical schools participated in the simulation. The mean age of participants was 24.3 ± 1.2 years, with a balanced gender distribution (53% female, 47% male). Ethnically, the cohort comprised 50% White, 27% Asian, 13% Black, and 10% students from other backgrounds, reflecting the diversity of medical trainees in the UK. All participants had completed at least one primary care rotation in a previous year, ensuring a baseline understanding of general practice and chronic disease management. However, the exposure to chronic disease progression and palliative care varied significantly, which may have influenced how participants engaged with different aspects of the simulation. All 30 students completed all phases of the study, ensuring full data availability (Table 3).

Regarding clinical experience, all students completed a structured primary care rotation, while 87% had an internal medicine placement, and 60% had undergone a surgical rotation. However, only 40% had direct experience managing chronic diseases, and 33% had exposure to palliative care, typically through hospice placements or hospital-based teams. Additionally, 60% had been involved in long-term patient follow-ups, providing them with insight into continuity of care challenges. A total of 47% had engaged in multidisciplinary case discussions related to chronic conditions, while 25% had shadowed geriatricians or long-term care specialists, indicating varying levels of familiarity with holistic disease management.

Beyond direct patient interactions, 80% of the participants had attended communication skills workshops, reinforcing their ability to apply patient-centred care principles in clinical settings. However, only 37% had prior experience with simulated patient exercises related to chronic disease progression, suggesting that, for many, this simulation was their first exposure to an immersive, long-term patient management experience. The varying levels of clinical exposure and prior experience with chronic conditions contributed to differences in learning outcomes, highlighting the importance of incorporating longitudinal disease management training into medical education.

### 3.2. Impact on Students’ Knowledge and Confidence

**Figure 2:**
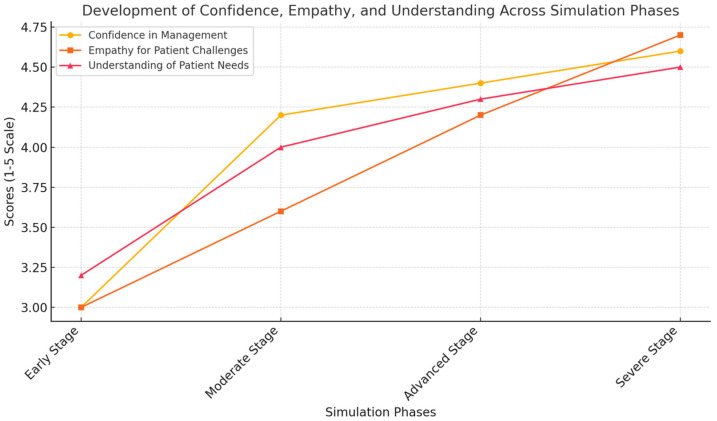
The development of confidence, empathy, and understanding across simulation phases. This figure highlights the progression of medical students’ confidence, empathy, and understanding of chronic disease management as they move through the four simulation phases. The gradual increase in scores reflects the effectiveness of the immersive learning experience, demonstrating how the exposure to simulated patient challenges enhances both clinical competence and emotional insight. The data underscore the importance of experiential learning in preparing future clinicians for long-term, patient-centred care.

The pre- and post-simulation survey data indicated significant improvements in students’ knowledge and confidence related to the management of chronic diseases and appreciation of the role of continuity of care (Table 4, Figure 2). Before the simulation, only 40% of students said they felt confident about their understanding of long-term disease trajectories; the mean confidence score was 2.8 ± 0.9 on a five-point scale. The percentage after the simulation increased to 87%, with a mean confidence score of 4.3 ± 0.6, reflecting a statistically significant improvement, with *p* < 0.01.

Students demonstrated very limited preliminary understandings of systemic issues contributing to challenges for chronically ill patients. For example, only 35% of students acknowledged how age-related changes, such as cognitive decline and limitations in mobility, influence the adherence to treatment and quality of life. Ninety-two percent of students post-simulation identified these factors as crucial, with the majority commenting that they had not realised how important they were.

As illustrated in the simulation, students also reported that their confidence in managing long-term conditions increased not only due to a theoretical understanding but because of a deeper awareness of how chronic disease unfolds over time. In particular, their understanding of age-related challenges—such as cognitive decline and mobility issues —was significantly strengthened. Post-simulation reflections indicated that students increasingly recognised the importance of the continuity of care, and they developed greater confidence in addressing systemic, non-clinical barriers, such as transportation difficulties and caregiver support needs. Empathy showed the greatest improvement, with many students commenting on how emotionally impactful the immersive experience was in helping them understand the psychological toll of chronic illness.

### 3.3. Students’ Experience During the Simulation

This simulation afforded students a unique lens through which to understand the progressive challenges their patients endured over two decades: Students reported little challenge in managing tasks such as organising medications or attending appointments in the early stages of the simulated disease, Years 0–5. As the simulation evolved, adding physical constraints—for example, weighted clothing to simulate frailty and goggles to mimic vision impairment—and cognitive tasks revealed the compounding challenges experienced by patients.

**Figure 3:**
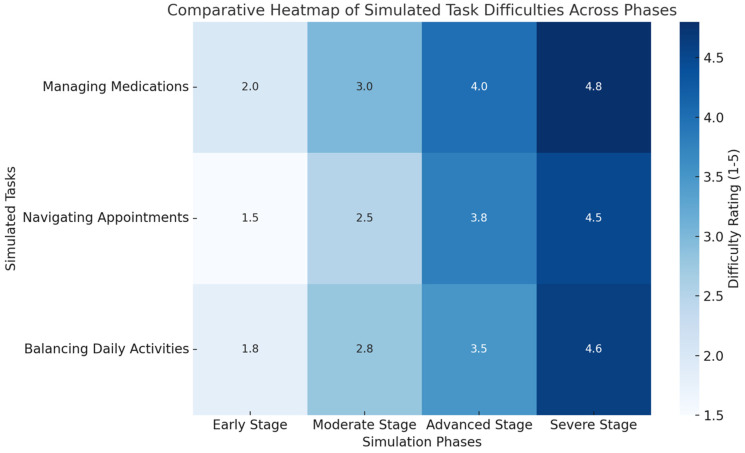
A comparative heatmap of simulated task difficulties across phases. This heatmap visualises the increasing difficulty of managing medications, navigating appointments, and balancing daily activities across the four simulation phases. Darker colours indicate greater challenges, emphasising the progressive nature of chronic disease impacts on daily life. The visualisation underscores the compounding burdens faced by patients, aligning with the experiential learning outcomes highlighted in this study.

At the Year 11–15 stage, 75% of students reported significant difficulty in the completion of tasks associated with daily living–simulated constraints, such as polypharmacy management and frequent doctor appointments. Students indicated that a chore that had previously been considered easy—for example, making an appointment or reading medication labels—became much more threatening with the addition of visual or cognitive contributory factors (Table 5, Figure 3). As one student reflected, “I hadn’t realised how much even minor impairments, like slightly blurred vision can make routine tasks so difficult”.

The final stage was Years 16–20, during which time all students reported a deep respect for the struggles associated with dependency upon caregivers and the psychological burden of lost independence. For many, this was the most influential phase; as one student commented, “It made me understand that chronic disease isn’t just about managing symptoms—it’s about helping patients navigate a life which changes completely over time”.

These changes were reflected in students’ reflections, such as their increased confidence in initial disease management, where they reported a better preparation for addressing patient concerns and treatment planning. During the moderate stage, students highlighted the significance of systemic barriers, such as transportation difficulties and physical limitations, which they had not previously considered in depth. In the advanced stage, students identified strongly with the challenges of polypharmacy and progressive impairments, expressing surprise at how small constraints like blurred vision affected routine tasks. By the severe stage, students demonstrated a greater awareness of the crucial role of caregivers and expressed a heightened ability to support patients with reduced independence and high dependency needs.

### 3.4. Post-Simulation Reflections

These themes were identified from the reflective journals as recurrent: the need to address nonclinical barriers, such as transportation and access to assistive devices, and proactive, long-term care planning. Most frequently, students described the simulation as transformative in shifting their perspective from episodic care to holistic, patient-centred management (Table 6). As was captured by one of the students: “This experience made me realise that treating the disease is only part of the job, understanding the person living with it is just as important”.

Students also suggested implementable ways of overcoming the barriers that were observed during the simulation: increasing access to home-based care services, improving communication between health professionals and their patients, and integrating caregiver support into regular care planning.

### 3.5. Quantitative Outcomes

The quantitative analysis of pre- and post-simulation survey responses showed marked improvements across multiple domains. Confidence in managing long-term disease trajectories increased from 2.8 ± 0.9 to 4.3 ± 0.6 (*p* < 0.01). The understanding of age-related challenges improved from 3.1 ± 1.0 to 4.5 ± 0.5 (*p* < 0.01). The recognition of the importance of the continuity of care rose from 3.2 ± 0.8 to 4.6 ± 0.4 (*p* < 0.01).

### 3.6. Qualitative Outcomes

**Figure 4:**
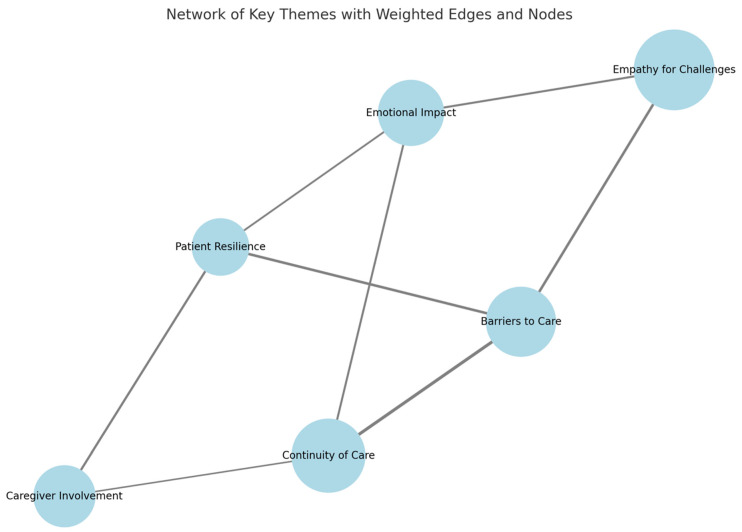
A weighted thematic network of chronic disease management: the prevalence and interconnections. This diagram illustrates the relationships between key themes identified in this study, with node sizes reflecting the prevalence of each theme in student reflections and edge thickness denoting the strength of connections between them. Larger nodes, such as “Empathy for Challenges” and “Continuity of Care”, indicate their central importance, while stronger edges highlight critical interdependencies, such as between “Barriers to Care” and “Continuity of Care”. This visualisation underscores the multifaceted and interconnected nature of managing chronic diseases effectively.

The qualitative analysis of the reflective journals and the debriefing sessions revealed a host of recurring themes, which included the following (Figure 4):

- Empathy for Progressive Challenges: students most often shared a new sense of empathy related to the progressive loss of independence associated with such illnesses.
- Barriers to Care: the major barriers to healthcare identified by the students were transportation, mobility restrictions, and declines in cognition.
- Continuity of Care: students continuously identified the need for continuity in care plans, which must be both evolving and patient-centred throughout the course of the patient’s disease.
- Psychological Effects that Patients Often Undergo: the simulation depicted, among other aspects, the psychological effects brought forth by chronic diseases, including feelings of isolation and frustration, which clinical settings usually fail to acknowledge.

## 4. Discussion

### 4.1. Key Results

The findings from this study illustrate the use of experiential learning as a means to enhance the learning of future physicians about the complexities related to chronic disease management over the long term. This pilot study provides evidence, for the first time, that Time Capsule Medicine can enrichen the current medical educational framework, as described by Nagel et al. [14].

The outcomes of the Time Capsule Medicine simulation highlight key areas for actionable improvements in medical curricula, particularly in fostering reflective practice, continuity of care, and holistic patient management. The significant increase in students’ confidence, empathy, and understanding of long-term disease trajectories suggests that integrating structured reflective activities, such as post-simulation debriefings and patient narratives, could reinforce experiential learning. These findings support the limited literature which highlights improved medical student knowledge and empathy attitudes when simulating the process of ageing [12]. Additionally, the simulation’s emphasis on the progressive nature of chronic diseases underscores the need for curricula to move beyond episodic care models and promote longitudinal patient follow-ups within training programmes [15]. Embedding holistic care principles, including addressing the social determinants of health and multidisciplinary collaboration, could further prepare students to manage chronic conditions in real-world settings.

Through the Time Capsule Medicine education framework, students simulated the decline of chronic illnesses over a period of 20 years and how those accumulate in tamping the everyday life of a patient with physical, cognitive, and emotional complications. This learning tool could be combined with longitudinal community-based clinical placements to further enrich the students’ understanding of chronic diseases [16]. Indeed, the results show significant increases for students in the knowledge of age-related changes, value of continuity, and, perhaps most importantly, their empathy. This is important as Time Capsule Medicine may also serve as a useful tool to reverse the decline in empathy commonly seen amongst senior medical students [17].

The Time Capsule Medicine simulation aimed to enhance medical students’ understanding of chronic disease progression, continuity of care, and patient-centred management. The baseline characteristics of the participants indicate that, while all had prior exposure to primary care, their experience with chronic disease management and palliative care was highly variable. This variation is reflected in the pre-simulation survey results, where the confidence in managing long-term disease trajectories was low (2.8 ± 0.9 on a 5-point scale). The fact that only 40% had direct experience managing chronic diseases suggests that the significant post-simulation improvement (4.3 ± 0.6, *p* < 0.01) was likely due to the structured exposure provided by the simulation. Similarly, the relatively low pre-simulation empathy scores (3.0 ± 0.9) improved markedly (4.7 ± 0.5, *p* < 0.01), reinforcing the idea that experiential learning is effective in developing a deeper understanding of patient challenges over time. This is corroborated in the literature, when medical students’ simulation of ageing improved their knowledge, attitudes, and empathy towards older people [12]

Students with prior exposure to long-term patient follow-ups (60%) and multidisciplinary case discussions (47%) may have had a better baseline appreciation for the complexities of chronic disease management. This could explain why students with prior chronic disease exposure reported a stronger ability to apply continuity-of-care principles post-simulation. However, the 33% of students with palliative care experience likely had a more developed understanding of end-of-life care needs, influencing their ability to navigate the later stages of the simulation, where patients became more dependent on caregivers. These insights align with the post-simulation reflections, where students identified the challenges of balancing the physical, emotional, and logistical dimensions of patient care over two decades.

Despite 80% of participants having attended communication skills workshops, only 37% had prior experience with simulated patient exercises related to chronic disease progression. This is particularly relevant given that the post-simulation qualitative feedback indicated that students found it challenging to manage the increasing complexity across disease stages, particularly in balancing polypharmacy, mobility restrictions, and cognitive decline. The findings suggest that structured, immersive role-playing—such as that provided by the Time Capsule Medicine framework—fills a crucial gap in traditional medical education, which often lacks dedicated training in longitudinal patient care. The data strongly support integrating experiential learning methods into medical curricula to bridge the disconnect between episodic clinical rotations and real-world chronic disease management.

### 4.2. Interpretations

Pre-simulation responses showed a limited understanding on the part of these students of the progression of chronic diseases, as well as issues unrelated to direct clinical care, such as transportation challenges, polypharmacy, and dependence on caregivers. This is in keeping with the literature, which finds that medical students often report little to no formal training in the management of chronic diseases [7]. Reflections at the conclusion of the simulation and survey results indicate quite a different outcome from the experience. Students identified that care must continue to shift away from the focus on the silo of symptom management and toward a holistic consideration of the larger social, emotional, and logistical context of a patient’s experience of illness. This positive change in attitude, likely from their participation in the Time Capsule Medicine study, is supported by research that identifies the ability to positively shift the medical students’ perspective towards the chronically ill through dedicated teaching [18].

The simulation brought out certain recurring themes in regard to the various challenges the students had to bear with while managing the chronic conditions. Of the many, one of the most striking balancing feats was required in their attending to the needs of patients along physical, emotional, and logistical dimensions [19]. In the process of the simulated disease, the students in the course reported a sense of overwhelm amidst a seemingly interactive decline in physical function and rise in the cognitive challenges and needs for social support. This mirrors real-life complexities that patients with chronic illnesses go through daily, and it is an issue that needs to be considered when training clinicians on how to prioritise these competing needs into a cohesive plan of care [20].

Students also struggled to cope with the psychological burden of disease chronicity. Many reflected upon the psychosocial effects of progressive illness, such as loneliness or frustration, for which they felt an underestimation existed. The capacity for empathy in regard to patients’ emotional experiences proved a seminal area of development among participants, which the literature shows to be correlated to clinical competency [21]. Indeed, Time Capsule Medicine may act as a tool that develops both clinical competencies and emotional intelligence.

The findings of this study carry serious implications for the design of medical curricula. Traditional medical education often focuses on acute care and episodic interventions with a limited emphasis on the longitudinal aspects of patient care [3]. This simulation-based approach represents a pragmatic and impactful method of addressing this gap by letting students experience the long arc of chronic illnesses in a controlled and reflective setting.

The changes noted in students’ empathy, acquaintance with processes of ageing, and awareness of non-clinical barriers add weight to the role of experiential learning in medical education [22,23]. This means that, in addition to the curriculum, programmes must equip them for continuity not only in care but also in long-term relationships with patients and the acceptance of the changing facets of their needs [24]. Additionally, reflective activities involve keeping journals and holding debriefing sessions, which support learning outcomes by encouraging students to synthesise experiences and then transfer them into actionable insights [25].

Building on this successful simulation, further work is planned to incorporate other elements that will make the exercise even more realistic and meaningful. For instance, the inclusion of further multidisciplinary input from social workers, occupational therapists, and caregivers would further enhance the investigation of the support mechanisms available for long-term disease management [26]. Case studies from different cultural backgrounds and varying socioeconomic statuses would allow for the recognition of how such factors affect patient experiences and access to healthcare [27].

Another area for development is the inclusion of patient narratives and testimonials. The literature shows that listening directly to people who have lived with chronic conditions can be a great asset to medical students [28] and thus help complement the simulated experience with the critical part of listening to and learning from patients.

These findings are of value beyond medical education, in informing healthcare practice. The challenges reported here by the students reflect systemic issues reported by real patients [29,30]—such as difficulty in accessing appointments, disjointed systems of care and support, and a lack of focus on mental health. Indeed, the management of chronic diseases requires a comprehensive, patient-centred strategy that emphasise continuity of care and address the patient’s changing needs over time [31].

This should also bring about the necessity for empathy and the building of healthier communication skills within the clinicians. Going forward, as health systems continue to be outcome- and efficiency-based, the human touch in care will be lost [32]. Those training programmes that will enable a clinician to perceive a patient as an individual with specific, long-term challenges—the people-centred approach—narrow this divide and make both patient satisfaction and healthy outcomes achievable [33].

Further research might also extend the simulation to include digital tools, such as electronic health records or telemedicine scenarios, addressing another underserved aspect of medical education [34]. Such a practical feature of administrative and technological management in long-term care would be added to the students’ learning and better prepare them for real-world practice.

### 4.3. Generalisability

The findings of this work have far-reaching implications for medical education, in particular in preparing students to cope with chronic disease in the long term. The design of the simulation and its underlying principles can be used in different educational settings, even though it was carried out in three medical schools in North London. The experiential learning approach used in Time Capsule Medicine is independent of high-tech tools, and hence it is possible to use it in medical schools of varying resource capabilities. The use of basic role-playing, facilitated reflection, and phased exposure to chronic disease progression means that students in different institutions can benefit from the approach independent of the specific healthcare infrastructure of their training setup.

Despite the controlled nature of the trial, there is more to be learned in terms of the aspects of generalisability. The trial group was a diverse group of final-year medical students with different exposures to primary care and chronic disease management in the lead-up to it. The impact of the intervention in various levels of training, such as specialist rotations or pre-clinics, is yet to be established. Future research would benefit from the application of this teaching methodology to different educational settings, such as general practice and hospitals. Cultural and healthcare system differences also impact the extent to which students engage with and learn from the challenges of chronic disease management. More studies with larger cohorts need to be undertaken to determine the efficacy of this model in various geographic locations, healthcare curricula, and student groups to determine if such increases in confidence, empathy, and patient-centred care can be replicated in other contexts beyond that of this initial trial.

### 4.4. Practical Applications and Scalability of Time Capsule Medicine Model

Incorporating basic technology, such as electronic health records (EHRs) and telemedicine scenarios, could enhance the realism and learning outcomes of the Time Capsule Medicine simulation by exposing students to digital tools commonly used in chronic disease management. EHR integration would allow participants to document and track a simulated patient progress over the 20-year timeline, reinforcing the importance of the continuity of care, medication reconciliation, and multidisciplinary communication. Similarly, telemedicine scenarios could simulate remote consultations with patients and caregivers, helping students develop skills in virtual chronic disease management, a growing aspect of modern healthcare. While not essential for the simulation’s core structure, these additions could provide valuable hands-on experience in digital healthcare delivery, preparing students for real-world primary care and specialist interactions.

The Time Capsule Medicine simulation can be adapted to various institutional settings by modifying its format based on available resources. Low-cost strategies include simple physical constraints (e.g., weighted backpacks and blurred vision sheets) and small-group role-playing instead of high-tech simulations. Virtual and hybrid models using online workshops, telemedicine scenarios, and digital case studies offer alternatives for institutions without physical training facilities. Integrating the simulation into existing curricula (e.g., primary care or geriatrics rotations) and leveraging patient narratives through recorded testimonials, mentorship programmes, or caregiver discussions ensures the accessibility and relevance across diverse settings (Table 7).

### 4.5. Limitations

While the findings demonstrate the effectiveness of the Time Capsule Medicine simulation in enhancing students’ understanding of chronic disease management, the generalizability of the results is limited by the small sample size (30 participants) and the study’s regional focus on three North London medical schools. Although the sample was diverse in terms of gender, ethnicity, and prior clinical experience, it may not fully represent medical students from different geographic, institutional, or healthcare system backgrounds.

Additionally, the high proportion of students with prior primary care exposure (100%) and communication skills training (80%) may have influenced the outcomes, as their baseline familiarity with continuity-of-care concepts might differ from that of students with predominantly hospital-based training. Moreover, there may be a potential self-selection bias, as students with a particular interest in chronic disease management may have been more inclined to participate. To mitigate these effects, future studies should consider expanding the participant pool across multiple medical schools, regions, and training levels to determine whether similar learning gains are observed in students with varying degrees of exposure and interest in chronic disease management. Additionally, incorporating a longitudinal follow-up could help assess the long-term impact of the simulation on clinical decision-making and patient-centred care approaches in real-world settings.

The absence of direct patient input is a missed opportunity for the first-hand inclusion of perspectives in the educational process, although it is considered appropriate for ethical reasons. Future aspects of this study could include narratives from patients or interviews as added components.

Although this study was able to prove that such time-lapse simulations are effective in improving learning regarding chronic disease management, its longer-term impact on clinical practice can only be guaranteed through further research. Longitudinal studies can identify how these kinds of experiential learning experiences influence students’ approaches to patient care as they go into professional roles [35]. Furthermore, the expansion in larger and more varied cohorts might allow one to use this as a base to better understand how cultural, socioeconomic, and institutional factors influence such simulations and their outcomes.

## 5. Conclusions

This study highlights the value of immersive, experiential learning in addressing key gaps in traditional medical education. Through a quantitative analysis, we observed significant improvements in students’ confidence, empathy, and understanding of chronic disease management following participation in the simulation. Qualitative findings further revealed a deepened insight into the continuity of care, emotional and functional patient challenges, and systemic barriers often overlooked in conventional training. Together, these results support the integration of time-lapse simulations into medical curricula as a means of fostering holistic, patient-centred approaches to long-term care. As healthcare systems increasingly emphasise continuity and compassion, educational innovations such as this can play a vital role in preparing future clinicians for the realities of lifelong patient relationships.

## Figures and Tables

**Table 1:** Structured phases of the “Time Capsule Medicine” simulation programme (The overview of key activities, learning objectives, and outcomes measured in each phase of the simulation programme).

Phase	Key Activities	Objectives	Outcomes Measured
Phase 1: Preparation	- Workshop on chronic disease progression and age-related changes. - Discussions on continuity of care and patient-centred approaches. - Pre-simulation surveys to assess baseline knowledge and attitudes.	- Introduce students to the concept of long-term disease trajectories. - Establish a baseline understanding of continuity of care.	- Pre-simulation knowledge and confidence scores. - Initial attitudes toward chronic disease management.
Phase 2: Immersive Simulation	- Role-playing exercise simulating a 20-year chronic disease journey in four five-year stages. - Physical constraints to mimic challenges such as mobility restrictions, vision impairment, and cognitive decline. - Documentation of observations at each stage, focusing on patient challenges and healthcare needs.	- Provide students with a first-hand understanding of long-term patient challenges. - Develop empathy for patients navigating chronic conditions.	- Changes in students’ perceptions of patient challenges. - Ability to document and reflect on evolving healthcare needs.
Phase 3: Reflection and Evaluation	- Facilitated debriefing sessions to discuss observations and share insights. - Completion of post-simulation surveys and reflective journals.	- Encourage students to synthesise their experiences and propose actionable strategies for continuity of care.	- Post-simulation knowledge and confidence scores. - Thematic analysis of reflective journals and feedback.

**Table 2:** Simulated stages of chronic disease progression: activities, challenges, and learning objectives.

Phase	Year Range	Key Activities	Simulated Challenges	Learning Objectives
Early Stage	0–5	Students simulated managing an initial diagnosis and initiating treatment plans.	Minimal functional impact; students focused on understanding disease basics and patient concerns.	Understand the patient’s emotional and practical challenges upon receiving a chronic diagnosis.
Moderate Stage	6–10	Role-playing involved navigating mobility challenges and intermittent symptoms while continuing treatment.	Weighted clothing to mimic frailty, scenarios for addressing adherence issues, and balancing daily tasks.	Build strategies for managing mid-stage disease progression and assisting patients with moderate limitations.
Advanced Stage	11–15	Students simulated managing complex medication regimens and significant physical impairments.	Blurred vision goggles, frequent doctor visits, and increasing reliance on assistive devices.	Recognise the cumulative challenges of polypharmacy, physical limitations, and emotional stress.
Severe Stage	16–20	Simulations emphasised dependency on caregivers and adapting care plans for cognitive decline.	Memory impairments, reliance on caregiver support, and structured medical routines.	Develop empathy and strategies for supporting patients with high dependency and reduced independence.

**Table 3:** Sociodemographic characteristics of participants (*n* = 30).

Characteristic	Value
Mean age (years)	24.3 ± 1.2
Gender	
Female	53% (*n* = 16)
Male	47% (*n* = 14)
Ethnicity	
White	50%
Asian	27%
Black	13%
Other	10%
Completed primary care rotation	100%
Completed internal medicine rotation	87%
Completed surgical rotation	60%
Prior chronic disease management exposure	40%
Prior palliative care exposure	33%
Involved in long-term follow-up care	60%
Attended communication skills workshops	80%
Prior experience with chronic disease simulation	37%

**Table 4:** Quantitative results—impact of simulation on student knowledge and confidence (Pre- and post-simulation scores reflecting changes in students’ confidence, empathy, and understanding of chronic disease management).

Metric	Pre-Simulation Score	Post-Simulation Score	Change
Confidence in managing long-term disease trajectories	2.8 ± 0.9	4.3 ± 0.6	+1.5 points
Understanding of age-related challenges (e.g., cognitive decline and mobility restrictions)	3.1 ± 1.0	4.5 ± 0.5	+1.4 points
Recognition of the importance of continuity of care	3.2 ± 0.8	4.6 ± 0.4	+1.4 points
Perceived ability to address non-clinical barriers (e.g., transportation and caregiver support)	2.9 ± 0.7	4.1 ± 0.6	+1.2 points
Empathy for patients with chronic illnesses	3.0 ± 0.9	4.7 ± 0.5	+1.7 points
Understanding of healthcare provider–patient dynamics	3.3 ± 0.8	4.5 ± 0.5	+1.2 points
Students who felt prepared to manage chronic conditions	35%	87%	+52%
Students who recognised age-related changes as critical in care planning	42%	92%	+50%

**Table 5:** Phase-specific metrics: changes in knowledge, confidence, and observations through simulation stages.

Phase	Metric	Pre-Simulation Score	Post-Simulation Score	Change
Early Stage	Confidence in initial disease management	3.0 ± 0.8	4.2 ± 0.5	+1.2
	Understanding of emotional challenges	3.2 ± 0.7	4.3 ± 0.6	+1.1
Moderate Stage	Confidence in managing mid-stage disease	2.9 ± 0.6	4.4 ± 0.4	+1.5
	Awareness of systemic barriers	2.8 ± 0.8	4.3 ± 0.5	+1.5
Advanced Stage	Empathy for progressive physical challenges	3.0 ± 0.9	4.6 ± 0.5	+1.6
	Confidence in polypharmacy management	2.7 ± 0.8	4.2 ± 0.5	+1.5
Severe Stage	Awareness of caregiver roles	3.1 ± 0.7	4.5 ± 0.4	+1.4
	Confidence in supporting high-dependency care	2.9 ± 0.9	4.4 ± 0.5	+1.5

**Table 6:** Thematic analysis of student reflections on simulation experience (Key themes from reflective journals, including empathy for progressive challenges, recognition of barriers, and proposals for care improvements.).

Theme	Description	Illustrative Student Quotes	Key Implications
Empathy for Progressive Challenges	Students developed a deeper understanding of the gradual, compounding difficulties faced by patients with chronic illnesses, including the loss of independence and emotional toll.	“I never realised how much something as small as blurry vision could disrupt someone’s daily life.”	Emphasises the need for holistic care that addresses not only physical symptoms but also emotional and logistical barriers.
Recognition of Barriers to Care	Participants identified systemic challenges, such as transportation difficulties, reliance on caregivers, and access to assistive devices, as significant barriers to effective care.	“It’s not just the disease—it’s how patients have to navigate everything around it that makes managing their health so hard.”	Highlights the importance of integrating support services and community resources into chronic disease management plans.
Emotional Impact on Patients	Students gained insight into the psychological toll of chronic diseases, including feelings of isolation, frustration, and dependency.	“Living with this condition for 20 years isn’t just about managing the disease—it’s about how it affects your entire identity.”	Suggests incorporating mental health support and counselling into routine care for patients with chronic conditions.
Importance of Continuity of Care	The simulation reinforced the value of sustained, adaptable care over decades to meet the evolving needs of patients.	“This made me realise that our role isn’t just to treat the disease—it’s to be there for the patient as their life changes.”	Encourages medical education to place greater emphasis on longitudinal care and patient-centred approaches.
Challenges in Balancing Priorities	Students found it difficult to address all aspects of a patient’s care, particularly when physical, cognitive, and emotional needs conflicted or compounded each other.	“It was overwhelming to think about balancing their medications, appointments, and mental health at the same time.”	Stresses the need for multidisciplinary care teams to address the multifaceted needs of patients with chronic illnesses.
Realisation of Patient Resilience	Students were impressed by the resilience and adaptability of patients despite significant challenges, fostering a greater appreciation for patient experiences.	“Even with all the difficulties, patients still find ways to get through their day—it’s inspiring and humbling.”	Suggests incorporating patient narratives and testimonials into medical training to complement simulations and foster respect for patient resilience.
Proposals for Care Improvements	Participants suggested actionable strategies, such as expanding home-based care, improving transportation support, and enhancing caregiver involvement in healthcare planning.	“We need to think beyond the clinic—patients need systems that make their lives easier, not more complicated.”	Encourages the development of innovative, patient-centred healthcare policies and interventions that address non-clinical barriers.

**Table 7:** Adaptation strategies for different resource levels.

Resource Level	Category	Adaptation Strategies
Low resource	Physical constraints	Use weighted backpacks, gloves, and blurred vision sheets instead of costly simulation tools.
	Role-playing and storytelling	Conduct small-group role-playing or use narrative-based storytelling to simulate disease progression.
	Curriculum integration	Embed within primary care, geriatrics, or internal medicine rotations to avoid extra infrastructure costs.
Moderate resource	Hybrid and digital models	Implement online workshops, virtual patient case studies, and telemedicine role-play exercises.
	Community-based learning	Partner with community health programmes, caregiver networks, and home-care services for real-world insights.
High resource	Advanced simulations	Utilise full-scale simulation labs with ageing suits, VR, or AI-driven patient cases to enhance realism.
	Interdisciplinary collaboration	Include input from social workers, occupational therapists, and caregivers in the learning process.
	Patient narrative alternatives	Use recorded testimonials, patient-led discussions, or mentorship programmes with long-term care providers.

## Data Availability

The datasets generated and/or analysed during the current quality improvement project are not publicly available due ethical reasons but are available from the corresponding author (W.J.) upon reasonable request.

## Appendix A. Student Forms

### Appendix A.1. Pre-Simulation Survey

Objective: To assess students’ baseline knowledge, confidence, and attitudes toward chronic disease management and continuity of care.

Instructions: Please rate the following statements on a scale of 1 to 5, where:

1 = Strongly Disagree, 2 = Disagree, 3 = Neutral, 4 = Agree, 5 = Strongly Agree.

**Statement**	**1**	**2**	**3**	**4**	**5**
- I understand the concept of chronic disease progression over an extended timeline.					
- I feel confident managing early-stage chronic conditions (e.g., newly diagnosed diabetes).					
- I understand the emotional challenges faced by patients receiving a chronic diagnosis.					
- I feel prepared to address functional challenges like mobility or vision loss in patients.					
- I am aware of the systemic barriers that patients encounter in accessing long-term care (e.g., transportation).					

Open-Ended Questions:

1. What do you expect to learn about chronic disease management from this simulation?
2. What do you think will be the most challenging aspect of managing long-term care for patients?
3. How do you currently approach patients with chronic conditions in your clinical practice?

### Appendix A.2. Post-Simulation Survey

Objective: To evaluate changes in students’ knowledge, empathy, and preparedness after participating in the simulation.

Instructions: Please rate the following statements on a scale of 1 to 5, where:

1 = Strongly Disagree, 2 = Disagree, 3 = Neutral, 4 = Agree, 5 = Strongly Agree.

**Statement**	**1**	**2**	**3**	**4**	**5**
- I now have a better understanding of how chronic diseases progress over two decades.					
- I feel confident managing mid-stage chronic conditions (e.g., mobility challenges or polypharmacy).					
- I have gained insight into the emotional and social challenges faced by patients with advanced conditions.					
- I feel prepared to manage patients with severe chronic conditions requiring caregiver support.					
- This simulation has improved my ability to adopt a holistic, patient-centred approach to long-term care.					

Open-Ended Questions:

1. What was the most valuable lesson you learned from this simulation?
2. How has your understanding of patient-centred care for chronic conditions changed after this experience?
3. What challenges did you face during the simulation, and how did you overcome them?

### Appendix A.3. Reflective Journal Prompts

Objective: To gather qualitative data on students’ experiences during each phase of the simulation.

4. Describe your experience managing a patient in the early stage of chronic disease progression. How did the patient’s emotional and practical challenges influence your approach?
5. Reflect on the challenges you encountered during the moderate stage of the simulation. What strategies did you find most effective in addressing these challenges?
6. What insights did you gain about managing polypharmacy and functional impairments during the advanced stage?
7. Discuss the key lessons from the severe stage regarding caregiver dependency and cognitive decline. How will these experiences shape your future practice?
8. Overall, how has this simulation influenced your perspective on continuity of care and the long-term impacts of chronic diseases?
